# Patterns of syphilis testing in a large cohort of HIV patients in Ontario, Canada, 2000–2009

**DOI:** 10.1186/1471-2334-13-246

**Published:** 2013-05-28

**Authors:** Ann N Burchell, Vanessa G Allen, Veronika Moravan, Sandra Gardner, Janet Raboud, Darrell HS Tan, Ahmed M Bayoumi, Rupert Kaul, Tony Mazzulli, Frank McGee, Peggy Millson, Robert S Remis, Sean B Rourke

**Affiliations:** 1 Ontario HIV Treatment Network, Suite 600, 1300 Yonge Street, Toronto, Ontario M4T 1X3, Canada; 2 Dalla Lana School of Public Health, University of Toronto, Toronto, Canada; 3 Public Health Laboratories, Public Health Ontario, Toronto, Canada; 4 Toronto General Research Institute, University Health Network, Toronto, Canada; 5 Division of Infectious Diseases, St. Michael’s Hospital, Toronto, Canada; 6 Centre for Research on Inner City Health, The Keenan Research Centre in the Li KaShing Knowledge Institute, St. Michael’s Hospital, Toronto, Canada; 7 Institute of Health Policy, Management and Evaluation, University of Toronto, Toronto, Canada; 8 Department of Medicine, University of Toronto, Toronto, Canada; 9 Mount Sinai Hospital, Toronto, Ontario, Canada; 10 AIDS Bureau, Ontario Ministry of Health and Long Term Care, Toronto, Canada; 11 Department of Psychiatry, University of Toronto, Toronto, Canada; 12 Department of Laboratory Medicine and Pathobiology, University of Toronto, Toronto, Canada

**Keywords:** Syphilis, HIV co-infections, Screening and diagnosis, Epidemiology

## Abstract

**Background:**

Since 2000, reported syphilis cases increased ten-fold in Canada, particularly among men who have sex with men (MSM) co-infected with HIV. We characterized temporal patterns of of syphilis testing in a large cohort of HIV patients in Ontario, Canada.

**Methods:**

We analyzed data from a multi-site cohort of people in HIV care from 2000 to 2009. Data were obtained from medical charts, interviews and record linkage with the syphilis test database at the Public Health Ontario Laboratories. We estimated the proportion that had syphilis testing at least once per year and the period and annual prevalence of reactive tests.

**Results:**

Among 4232 participants, the annual proportion tested rose from 2.7% (95%CI 1.9, 3.5) in 2000 to 54.6% (95%CI 52.9, 56.3) in 2009. Testing was most common for participants who were men who have sex with men (MSM), aged <30, recently diagnosed with HIV, were antiretroviral treatment naive, had routine HIV lab testing at least twice in that year, or tested for syphilis in the preceding year. The proportion with at least one reactive test in 2000–09 was 21.0% (95%CI 19.4, 22.7) for MSM, 5.3% (95%CI 3.3, 7.4) for non-MSM males, and 2.6% (95%CI 1.2, 4.0) for women. Among MSM, the annual prevalence of reactive syphilis tests with high RPR titre (≥1:16) peaked at 3.8% in 2009.

**Conclusions:**

The burden of syphilis co-infection rose considerably among HIV-positive MSM, such that by 2009, at least 1 in 5 men had laboratory evidence of current or past infection. Interventions may be needed to boost syphilis testing to achieve goals set by guidelines even in settings with universal health care.

## Background

After years of decline, syphilis has re-emerged as a serious health burden among urban men who have sex with men (MSM) in industrialized countries [1]. In Canada, notifiable cases of infectious syphilis rose from 0.6 per 100,000 in 2000 to 5.0 per 100,000 in 2009 [2] and outbreaks have been identified in urban centres across the country [1,3-5]. Most reported cases are among MSM, many of whom are co-infected with HIV. In Ontario, most reported syphilis cases have been in the two largest cities, Toronto and Ottawa, with concurrent HIV infection present in 45% of cases; HIV prevalence might be even higher given that HIV status was unknown for men who were not tested for HIV or tested anonymously (personal communication, Michael Whelan, Public Health Ontario, April 30, 2012). In contrast, HIV prevalence is 18% and 11% among MSM in Toronto and Ottawa, respectively [6,7] suggesting that HIV-positive men are over-represented in syphilis transmission networks. For men co-infected with HIV, the response to syphilis treatment can be suboptimal, [8] the development of neurosyphilis may be accelerated, [9] treatment decisions are more complex, and HIV infectiousness may be enhanced [10]. Prompt syphilis testing and treatment is important to prevent potential clinical sequelae and also to limit ongoing syphilis and HIV transmission. However, to our knowledge, patterns of syphilis testing among people with HIV have yet to be explored in Ontario.

North American guidelines for syphilis testing among HIV-positive MSM are generally consistent in recommending at least annual testing. Canadian guidelines recommend that people diagnosed with HIV undergo further screening for syphilis and other sexually transmitted infections (STIs); that STI risk be discussed routinely with HIV patients at each visit; and that MSM with STI risk, regardless of HIV status, undergo syphilis screening at least yearly [1]. U.S. guidelines recommend that sexually active persons with HIV have routine screening for curable STIs, including syphilis, at least annually [11].

Our goal was to describe syphilis testing patterns among HIV patients in care in Ontario, Canada, a setting which has publicly-funded universal access to medically necessary health-care services. Specific objectives were to estimate the proportion undergoing syphilis testing at least annually; to characterize persons who were testing compared to those who were not; and to estimate the prevalence of past or current syphilis.

## Methods

We analyzed data from the Ontario HIV Treatment Network Cohort Study (OCS). The study design has been described previously [12]. Briefly, the source population consists of people aged 16 and older diagnosed with HIV infection and who receive medical care in Ontario at hospital-based specialty HIV outpatient clinics and family practice units and community-based primary care physician practices. In the Ontario setting, most HIV care is provided by a small number of specialized practitioners who offer integrated, interprofessional HIV outpatient care, as well as a a significant amount of primary care (Jean Bacon, OHTN, personal communication, January 28, 2013). The 12 participating clinics served over three-quarters of HIV patients in the province [12]. Participation in the OCS was voluntary. All provided written, informed consent. Clinical data obtained as part of participants’ routine health care were abstracted from clinic records. From 1995–2007, participants self-completed a questionnaire at enrolment. Since 2008, they underwent an annual interview. The study protocol, research instruments and forms received ethical approval from the University of Toronto Human Subjects Review Committee and from the individual study sites.

### Syphilis testing and diagnosis

We obtained syphilis testing data through record linkage with the Public Health Ontario Laboratories (PHOL), the sole provider of syphilis testing in Ontario. Serology is the primary diagnostic methodology used for syphilis testing. Prior to August 2005, the testing algorithm consisted of the non-treponemal Rapid Plasma Reagin (RPR) test as a screen, followed by confirmatory test methodologies including the *Treponema pallidum* particle agglutination test (TPPA) and the fluorescent treponemal antibody absorbed (FTA-ABS) test. Subsequently, the testing algorithm changed to screening with a treponemal test, the Abbott chemiluminescent immunoassay, followed by confirmatory testing with the RPR, TPPA, and FTA-ABS tests [13]. The latter algorithm has greater sensitivity to detect early syphilis cases.

We classified the requesting institution for each syphilis test as (1) the HIV clinic at which the patient was participating in the OCS; (2) a designated sexual health clinic administered by local public health units; or (3) all other health care providers combined.

We classified syphilis test results as (1) reactive, defined as a reactive trepomenal test, regardless of RPR titre; (2) non-reactive; or (3) undetermined. Given this laboratory-test-based definition, a case with any test reactivity included recent incident cases and persons with evidence of past, treated syphilis infection. Furthermore, we distinguished reactive cases as “acute” or “non-acute”, defining acute as having an RPR titre of 1:16 or greater, suggestive of infectious syphilis. In general, infectious syphilis is considered to be more likely when RPR titres are 1:32 or greater [1]. However, we used a more liberal cut-point for our definition of acute syphilis, a titre of 1:16, because serologic responses may be altered in HIV co-infected patients, and can include false-negative or delayed seropositivity [11,14]. We reviewed medical chart data from participating HIV clinics for information on syphilis stage, where available, for all participants who had at least one acute syphilis test in 2000–09.

### Analysis

We analyzed data available as of December 2010, at which time 5,644 participants had enrolled. PHOL syphilis test records were available for the 2000–09 period. Therefore, we restricted the analysis to participants under prospective follow-up during that time (952 excluded) and for whom there was successful record linkage to the HIV viral load database at the PHOL, suggesting that any syphilis testing done by any care provider in Ontario should have been observable (460 excluded). The final analytical sample size was 4,232 participants. We conducted all statistical analyses using SAS version 9.3 (SAS Institute, Inc., Cary, North Carolina). All p-values were two-sided and statistical significance was determined using the traditional p-value of < 0.05.

We used descriptive statistics to characterize participants included in the analysis, the proportion that had at least one linked syphilis test, and the type of requesting institution. We estimated the proportion of syphilis tests that were carried out concurrently with an HIV viral load test, defined as the same date of specimen collection or the same date of specimen testing, plus or minus three days. We compared proportions between MSM, other males, and females, and over calendar years, using logistic regression within a generalized estimating equations (GEE) framework to account for repeated tests per person.

Next, we calculated annual rates of syphilis testing, defined as the proportion that underwent testing at least once in a calendar year while under observation in the cohort. Participants were considered under observation starting at the year they enrolled in the OCS, or the year 2000, whichever was later. They remained under observation for each subsequent year until their last year of follow-up (i.e., year of death, loss-to-follow-up, or 2009 for those still under active follow-up as of December 31, 2009). We report proportions with 95% confidence intervals (CI).

To assess predictors of testing, we estimated the ratio of prevalence proportions rather than odds ratios because the odds ratio can greatly overestimate the ratio of proportions for a common outcome. We used PROC GENMOD and Poisson regression using GEE with an auto-regressive correlation structure. This approach accounts for repeated measures by adjusting standard errors for the actual number of participants and it is also recommended for calculation of ratios of proportions or prevalence ratios [15]. For participants who died, we used an offset to censor their observation at the date of death, such that they only contributed a fraction of a person-year during the year of death. Participants who entered or exited the study in a calendar year due to consent or loss-to-follow-up contributed a full person-year because we assumed that provincial in- or out-migration would have been trivial, and our record linkage with the PHOL ensures that we would have detected any testing conducted in Ontario in that year.

We analysed patterns of repeat syphilis testing in the subset of patients with at least two years of follow-up, but stratified our analysis by past syphilis test result to distinguish screening for new infection from follow-up testing after syphilis diagnosis and treatment. First, we calculated inter-test intervals among patients whose previous test(s) was/were non-reactive or who had only non-acute reactive tests for a period of at least two years; tests for differences in inter-test intervals were carried out using linear regression with GEE to account for repeated measures person among those who tested three or more times. Second, we examined patterns of repeat testing among cases who had their first reactive test in 2006–09, and evaluated follow-up testing in line with clinical guidelines that recommend repeat syphilis testing of newly-identified cases at (possibly 1), 3, 6, 12, and 24 months following treatment [1,11].

Finally, we used descriptive statistics and 95%CI to estimate the period and annual prevalence of a diagnosis of syphilis, stratified by patient sex and MSM status. In all prevalence estimates, untested participants remained in the denominator because all had the potential to have been screened for and diagnosed with syphilis. Our approach is analogous to population-based rates of reportable STI incidence in public health surveillance, which include the total population in the region in the denominator, regardless of their testing status.We defined period prevalence of past or current syphilis as the proportion that underwent testing and had at least one reactive test result over the 2000–09 period; this approximates lifetime prevalence. We defined annual prevalence of a diagnosis of past or current syphilis as the proportion of participants under observation in a given calendar year who had at least one reactive test result in that year; for those who had reactive and non-reactive results in a single year, we classified them as a prevalent case. We then calculated the annual prevalence of a diagnosis of acute syphilis, defined as the proportion of participants who underwent testing and had at least one acute reactive test in that year. We also conducted a test-based analysis to compare reactivity rates by requesting institution type; we used a proportional odds model with GEE for the ordinal response categories of non-reactive, reactive non-acute, and acute.

## Results

On average, participants were male (86%), aged 44 years, and reported White race (67%). The median year of HIV diagnosis was 1995 (IQR 1990–2001) (Table 1). At enrolment, the mean CD4 cell count was 455 cells/mm^3^ (SD 266) and the median viral load was 1.4 log_10_ copies/mL.

**Table 1:** Characteristics of 4,232 HIV-positive participants in the OHTN Cohort Study, 2000-2009

	**n**	**%**
Female	608	14.4%
HIV exposure category		
MSM	2647	62.5%
MSM-IDU	207	4.9%
IDU	319	7.5%
Endemic	330	7.8%
Heterosexual	447	10.6%
Other risk	146	3.4%
Unknown risk	136	3.2%
Region of Ontario		
Toronto	2680	63.3%
Ottawa	508	12.0%
Other	1044	24.7%
Ethnicity		
White	2854	67.4%
Black/African	409	9.7%
Multiple	318	7.5%
Aboriginal	247	5.8%
Other	235	5.6%
Unknown	169	4.0%
Any HIV antiretroviral medication during follow-up	3739	88.3%
Mean age at baseline* (SD)	43.5	(9.6)
Median year of HIV diagnosis (IQR)	1995	(1990–2001)
Median # months of prospective follow-up (IQR)	22	(13–89)
Mean CD4 cell count/mm3 at baseline* (SD)	455	(266)
Median log10 viral load at baseline* (IQR)	1.4	(1.4-3.7)

MSM, men who have sex with men. IDU, injection drug user. SD, standard deviation. IQR, interquartile range.

* Baseline = later of January 1, 2000 or enrolment date.

### Syphilis testing

A total of 7,313 syphilis tests were linked to the 4,232 participants, with the majority (81.5%, 3,451/4,232) having undergone testing for syphilis at least once in the 2000–09 period. This proportion was highest for MSM (83.4%, 2,422/2,903) but only slightly more so than among women (78.3%, 476/608) and non-MSM males (74.6%, 461/618). Among the 3,451 testers, the median number of tests was 2 (IQR 1–4).

The majority of tests (85.6%, 6,261/7,313) were ordered by the HIV clinic at which the participant had enrolled in the OCS. Tests were less commonly ordered by sexual health clinics (5.0%, 369/7,313 tests) and other health care providers, e.g., non-HIV specialist family physicians (9.3%, 683/7,313 tests). MSM testers were more likely to have undergone testing at sexual health clinics (11.1%) compared to non-MSM males (5.8%) or females (4.5%) (chi-squared test, p=.0001).

Overall, 77.2% (5,646/7,313) of syphilis tests were ordered concurrrently with an HIV viral load. This proportion did not vary appreciably between MSM, other males, and females (data not shown). However, there was a temporal trend such that the proportion increased from 44.4% in 2000 to 79.1% in 2009 (p<0.0001).

### Annual proportion tested

**Figure 1:**
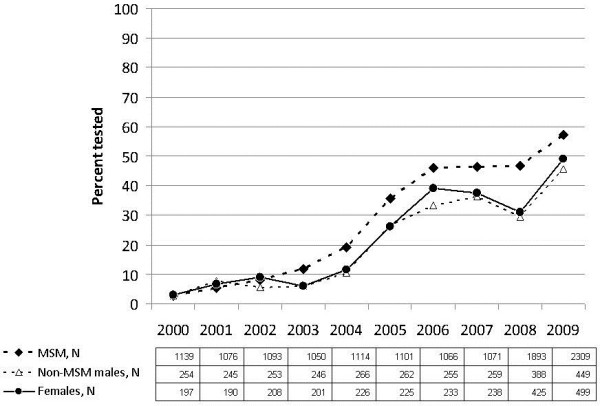
Annual proportion tested for syphilis among HIV-positive participants in the OHTN Cohort Study, by MSM status.

The annual proportion tested rose from 2.7% (95%CI 1.9, 3.5) in 2000 to a peak of 54.6% (95%CI 52.9, 56.3) in 2009, with most of the increase occurring in the first half of the decade and minimal changes occurring after 2005 (Figure 1, Table 2). The rise in testing was observed among all participants. In virtually every calendar year, testing was most common among MSM. By 2009, the proportion testing annually was 57.2% (95%CI 55.2, 59.2) among MSM, 49.1% (95%CI 44.7, 53.5) among females, and 45.7% (95%CI 41.0, 50.3) among non-MSM males.

**Table 2:** Annual proportion tested for syphilis among HIV-positive participants in the OHTN Cohort Study, 2000-2009

	**n observation**	**% tested**	**Unadjusted ratio of proportions (95%CI)**	**Adjusted ratio of proportions (95%CI)**
Calendar year				
2000	1605	2.7		
2001	1524	6.0		
2002	1566	7.7		
2003	1507	10.2		
2004	1627	16.8		
2005	1616	32.6		
2006	1580	43.3		
2007	1592	43.6		
2008	2767	42.3		
2009	3324	54.6		
*Each additional calendar year (a)*				
2003 cf 2002			1.59 (1.54, 1.69)	1.53 (1.48, 1.58)
2005 cf 2004			1.39 (1.36, 1.42)	1.32 (1.30, 1.35)
2009 cf 2008			1.05 (1.03, 1.08)	0.98 (0.96, 1.01)
STI risk status				
MSM	12,912	31.7	1.39 (1.27, 1.53)	1.20 (1.11, 1.30)
Non-MSM males	2,877	22.6	Referent	Referent
Females	2,642	26.2	1.17 (1.03, 1.32)	1.03 (0.93, 1.14)
Unknown	277	47.3		
Region				
Toronto	10,014	31.7	1.19 (1.11, 1.27)	0.83 (0.78, 0.88)
Ottawa	2,527	26.9	0.89 (0.80, 0.99)	0.70 (0.64, 0.77)
Other	6,167	27.8	Referent	Referent
Age (b)				
<30 years	576	41.8	Referent	Referent
30+ years	18,132	29.4	0.72 (0.64, 0.81)	0.89 (0.81, 0.97)
Race/ethnicity				
White	13,322	28.6	Referent	
Black/African	1,463	28.9	1.01 (0.90, 1.13)	
Aboriginal	1,092	32.6	1.13 (1.00, 1.27)	
Other	2,363	33.4	1.17 (1.08, 1.27)	
Unknown	468	42.5		
Nature of care provided at OCS clinic				
Primary	4,242	44.5	1.74 (1.64, 1.84)	1.34 (1.27, 1.41)
Tertiary	14,466	25.5	Referent	Referent
Diagnosed with HIV in current or preceeding year (b)				
No	18,330	29.1	Referent	Referent
Yes	378	63.5	1.88 (1.73, 2.04)	1.29 (1.19, 1.41)
Syphilis testing in previous year (b)				
Not tested	13,995	18.3	Referent	Referent
Non-reactive	3,755	62.0	4.90 (4.69, 5.12)	1.75 (1.66, 1.84)
Reactive	888	73.6	5.45 (5.18, 5.73)	2.01 (1.89, 2.15)
Test result undetermined (c)	70	35.7		
Initiated HIV antiretoviral treatment (b)				
Yes	17,620	29.2	0.75 (0.68, 0.83)	0.92 (0.86, 0.99)
No	1,088	38.6	Referent	Referent
Number of routine HIV tests in year (b, d)				
None	451	4.2	0.18 (0.11, 0.29)	0.23 (0.14, 0.40)
One	1186	23.4	Referent	Referent
Two or more	17071	30.9	1.32 (1.20, 1.45)	1.27 (1.16, 1.40)

Each observation represents a single calendar year of active follow-up. We calculated ratios of proportions using Poisson regression within a generalized estimating equation framework to account for repeated observations. The within-subject correlation coefficient was 0.40 in an intercept-only model. The adjusted model included all covariates shown except ethnicity.

(a) There were statistically significant quadratic effects of calendar year. Beta coefficients for the linear and quadratic terms were 1.89 (95%CI 1.79, 2.01) and 0.966 (95%CI 0.962, 0.971) in the unadjusted model, and 1.84 (95%CI 1.74, 1.94) and 0.964 (0.960, 0.969) in the adjusted model, respectively.

(b) Time updated each calendar year.

(c) Reasons for unavailable test results in the preceding year included that the specimens were not tested or results were pending.

(d) The number of routine HIV tests in the year was defined as the maximum of the total number of HIV viral load or the total number of CD4 cell count tests in a calendar year, whichever was greater. It is a proxy measure of the frequency of contact with HIV care.

Testing was most common among people in their twenties (Table 2); analysis of age as a continuous variable demonstrated little variation in testing rates among those aged 30 or older. Testing was more common among participants who had enrolled in the OCS at primary care clinics, relative to tertiary, hospital-based HIV outpatient clinics. Syphilis testing was most likely for those who were diagnosed with HIV in the same or the preceding year, for those who had not yet initiated antiretroviral therapy, and for those who had undergone routine HIV laboratory testing at least twice in that year. Finally, we observed that participants who were tested for syphilis in one year were approximately twice as likely to test in the subsequent year, and more so if they had a reactive test in the preceding year.

We repeated the analysis in a subsample restricted to MSM, and observed no substantial differences in the correlates of annual testing (data not shown). We also repeated the analysis excluding syphilis testing done at such times may reflect a battery of baseline testing for HIV patients rather than routine syphilis testing done as part of ongoing HIV care, and observed little change to the estimated proportions of annual testing (data not shown).

### Frequency of syphilis testing

Among patients who were tested for syphilis at least once and were followed for at least two years, 74.6% (671/900), 62.6% (119/190) and 66.9% (107/160) were tested two or more times among MSM, other males, and women, respectively. Among repeat testers who had previously tested negative or had non-acute reactive results for a period of at least two years, the median inter-test interval was 6.0 months (IQR 3.1-11.0). This interval was shortest among MSM (median 5.5, IQR 3.0-10.1) compared to other males (median 10.4, IQR 6.2-13.4) and women (median 11.2, IQR 5.6-14.0); however, the differences were not statistically significant, nor was there evidence of a temporal trend (data not shown).

Follow-up testing after syphilis diagnosis and treatment is recommended by clinical guidelines; [1,11] therefore we examined patterns of repeat testing in a subset of 199 newly-identified cases who had their first reactive test in 2006–09. Including only cases who had sufficient follow-up in the denominator, we observed repeat syphilis testing for 20% (40/199) in the subsequent 1–31 days, 39% (75/193) in the subsequent 32–93 days, 43% (74/172) in the subsequent 94–183 days, 56% (66/119) in the subsequent 184–365 days, and 55% (38/69) in the subsequent 366–731 days following the first reactive result.

### Prevalence of diagnosed syphilis

Among participants under observation in 2009, 21.0% (95%CI 19.4, 22.7) of MSM underwent syphilis testing and had at least one reactive test in 2000–09. The corresponding period prevalence estimates for a diagnosis of past or current syphilis were 5.3% (95%CI 3.3, 7.4) for non-MSM males, and 2.6% (95%CI 1.2, 4.0) for females.

We reviewed medical chart data for information on syphilis stage for the 228 participants who had at least one acute syphilis test. For the majority (57%, 130), there was insufficient information to stage. Among the remaining 98 cases, 87% were diagnosed with infectious syphilis (primary, secondary, early latent); 10% were diagnosed with neurosyphilis, 2% with late latent syphilis or latent syphilis of unknown duration; and 1% were diagnosed with ocular syphilis.

In each calendar year from 2000 to 2009, we calculated the annual prevalence of a diagnosis of syphilis (Table 3). The predominant burden of reactive tests, both acute and non-acute, was among MSM and this increased over the decade. Conversely, the proportion with reactive results was far less among non-MSM males or females, and virtually no acute results were detected in those patient populations.

**Table 3:** Annual prevalence of reactive syphilis results among HIV-positive participants in the OHTN Cohort Study, 2000-09

	**N under observation**	**Not tested**		**Non-reactive**		**Reactive**		**Reactive & acute (a)**	
		**N**	**%**	**N**	**%**	**N**	**%**	**N**	**%**
**MSM**									
2000	1139	1108	97.3	19	1.7	6	0.5	1	0.1
2001	1076	1019	94.7	35	3.3	4	0.4	1	0.1
2002	1093	1006	92.0	67	6.1	12	1.1	5	0.5
2003	1050	927	88.3	83	7.9	36	3.4	15	1.4
2004	1114	899	80.7	162	14.5	47	4.2	17	1.5
2005	1101	708	64.3	330	30.0	61	5.5	15	1.4
2006	1066	576	54.0	384	36.0	100	9.4	12	1.1
2007	1071	575	53.7	392	36.6	103	9.6	7	0.7
2008	1893	1007	53.2	631	33.3	246	13.0	47	2.5
2009	2309	988	42.8	918	39.8	400	17.3	88	3.8
P-value for time trend						P < 0.0001		P < 0.0001	
**Non-MSM Males**									
2000	254	247	97.2	5	2.0	1	0.4	0	0
2001	245	226	92.2	14	5.7	2	0.8	0	0
2002	253	239	94.5	11	4.3	3	1.2	1	0.4
2003	246	231	93.9	11	4.5	4	1.6	1	0.4
2004	266	238	89.5	28	10.5	0	0	0	0
2005	262	192	73.3	67	25.6	3	1.1	0	0
2006	255	170	66.7	75	29.4	10	3.9	1	0.4
2007	259	165	63.7	84	32.4	10	3.9	0	0
2008	388	274	70.6	102	26.3	12	3.1	0	0
2009	449	244	54.3	192	42.8	11	2.4	0	0
P-value for time trend						P = 0.0002		P = 0.4443	
**Females**									
2000	197	191	97.0	5	2.5	0	0	0	0
2001	190	177	93.2	10	5.3	0	0	0	0
2002	208	189	90.9	19	9.1	0	0	0	0
2003	201	189	94.0	12	6.0	0	0	0	0
2004	226	200	88.5	26	11.5	0	0	0	0
2005	225	166	73.8	59	26.2	0	0	0	0
2006	233	142	60.9	89	38.2	1	0.4	0	0
2007	238	149	62.6	88	37.0	1	0.4	0	0
2008	425	293	68.9	125	29.4	7	1.6	0	0
2009	499	254	50.9	234	46.9	11	2.2	0	0
P-value for time trend						P < 0.0001		n/a	

Percentages shown are row percents. For participants who underwent testing more than once in a single calendar year and had both reactive and non-reactive results, we classified their results as reactive. P-values for calendar year time trends were calculated using logistic regression with GEE.

(a) Acute reactive results were defined as a reactive trepomenal test and RPR titre greater than or equal to 1:16.

Finally, we compared the test-based reactivity rates according to the type of requesting institution. There was an association that approached statistical significance for syphilis tests ordered at sexual health clinics to be reactive (35.7%, 132/369) or acute (8.9%, 33/369), compared to tests ordered by other types of health care providers (reactive: 25.6%, 1779/6944; acute: 4.3%, 300/6944; p=0.07 proportional odds regression with GEE).

## Discussion

We analysed data from persons in HIV care in Ontario, Canada in 2000–09 and observed that the annual proportion tested for syphilis rose from 2.7% in 2000 to a peak of 54.6% in 2009, with most of the increase occurring in the first half of the decade and minimal changes occurring after 2005. The observed rise in testing is encouraging, yet rates still remain far below international guidelines for screening frequency. The predominant burden of reactive tests was among MSM and this increased substantially over the decade.

Testing was most common among MSM, people aged <30 years, those who had tested in the preceding year, or those with more HIV lab tests in that year. We consider the latter variable to be a proxy measure of clinic visit encounters, such that more HIV care visits provided had more opportunities for syphilis test orders. Similarly, the main predictors of chlamydia and gonorrhea testing were younger age and more frequent visits per year among HIV-positive men in care at a Baltimore HIV clinic [16]. An important correlate of annual syphilis testing was having been tested in the preceding year, suggesting that those who test are likely to test again. Certainly, clinical guidelines advise that persons diagnosed with syphilis undergo follow-up testing at 3, 6, 12 and 24 months [1].

Most syphilis tests were ordered by patients’ HIV care providers and many were ordered concurrently with HIV viral load testing, suggesting that HIV clinics are a natural target for further improvements in syphilis screening. Health care providers are important prompters of syphilis testing and many MSM already have a practice of routine testing. In a recent online survey in Toronto and Ottawa, the most commonly mentioned reason for syphilis testing among HIV-positive MSM was “it is part of my regular testing pattern” (54%), followed by “my health care provider suggested it” (27%) (Frank McGee, AIDS Bureau, Ontario Ministry of Health and Long Term Care, personal communication July 18, 2012).

Our results suggest that the lifetime prevalence of syphilis is at least 1 in 5 among HIV-positive MSM in Ontario, and that 3.8% had evidence of acute syphilis infection in 2009, as indicated by RPR titres of 1:16 or higher. Our findings are consistent with clinic-based studies of HIV-positive persons conducted elsewhere, for which occurrence of new syphilis infection is typically 3-5%, with the vast majority among MSM [17-20]. The increased proportion of acute syphilis test results imply a substantial and growing burden for clinical management and treatment. In preliminary analyses among MSM in our cohort, we estimated that the incidence of the first syphilis diagnosis was 4.0 per 100 person-years (95%CI 3.0, 5.2), and the rate of re-diagnosis based on a four-fold rise in RPR titre was even higher at 4.8 per 100 person-years (95% 3.7, 5.5) [21]. Our team is currently conducting an in-depth analysis of risk factors for incident syphilis and re-diagnoses which will be the subject of a future paper.

Study limitations include our use of a non-random sample. Participating clinics serve over three-quarters of HIV patients undergoing viral load testing in the province [12]. Nevertheless, compared to Ontario surveillance data, [22] participants were more likely to be MSM or MSM-IDU but less likely to have been infected via heterosexual activity. We were unable to determine whether syphilis testing appropriately reached those at greatest risk of syphilis because sexual behaviour was unmeasured during the period of study. It is unknown whether testing was routine, was prompted by signs or symptoms, sexual exposures, contact tracing, or greater awareness of syphilis outbreak reports. The prevalence analysis was susceptible to selection bias because only participants who underwent testing could be included in the numerator. Therefore, prevalence was underestimated because true cases of syphilis would have been undetected for asymptomatic, unscreened cases. Finally, prevalent cases include both recent acquisitions and past syphilis infections.

## Conclusions

The rise in testing in Ontario in the first half of the decade follows closely the surveillance reports of new syphilis outbreaks, with the vast majority of cases among MSM, many of whom were co-infected with HIV; incidence of reported syphilis rose from 0.4 per 100,000 in 2000 to 4.0 per 100,000 in 2004, followed by a drop to 2.9 per 100,000 in 2005, and then a continued rise to a peak of 6.0 per 100,000 in 2009 [1-5]. However, since 2005 the annual testing coverage plateaued in the face of an ever-increasing burden of infection, and was still considerably lower than what would be desirable based on STI guidelines. There is an urgent need for novel interventions to boost testing. Similar suboptimal testing has been observed for other pathogens among HIV patients. In an HIV clinic with a high degree of awareness of STI epidemics in Baltimore, Maryland, the introduction of U.S. CDC guidelines for chlamydia and gonorrhea testing resulted in only a moderate increase in testing for these pathogens from 12% to 33% tested per year [16,23]. In general, a Cochrane systematic review determined that the distribution of printed educational materials, including clinical guidelines, generally produced meager benefits in the health care process [24]. Although guidelines serve an important purpose in clarifying best practice and reach a wide audience at little cost, they are insufficient to produce maximal uptake.

Operational interventions may be necessary to increase syphilis testing rates. For example, standing orders for pneumococcal vaccine are more effective at increasing rates of vaccination compared to usual practice, even more so than computerized reminders [25]. There is a growing body of research which suggests that clinic-based interventions can be effective in improving syphilis screening coverage, frequency, and case detection among HIV-positive MSM [26-29]. Even for studies conducted in specialist HIV clinics, where physician knowledge of sexual health and STI issues was presumably high, the interventions resulted in improvements, suggesting potential benefit even for STI-experienced providers, and even greater potential for settings with low or infrequent STI screening [28]. We recommend that future efforts to improve timely detection and treatment of syphilis in this population include systematic and operational changes to health care practice, such as clinic-based interventions.

## Competing interests

The authors declare that they have no competing interests.

## Authors’ contributions

ANB conceived the study, coordinated the data collection and analysis, and drafted the manuscript. VA supervised syphilis testing at the PHOL and guided test interpretation, with support from DT, RK, and TM. VM carried out the statistical analysis with guidance from SG and JR. SBR, AMB, FM, PM and RSR advised on interpretation of the findings within the greater context of the syphilis-HIV co-epidemic in Ontario, Canada. All authors read and approved the final manuscript.

## Pre-publication history

The pre-publication history for this paper can be accessed here:

http://www.biomedcentral.com/1471-2334/13/246/prepub
